# AXL targeting reduces fibrosis development in experimental unilateral ureteral obstruction

**DOI:** 10.14814/phy2.14091

**Published:** 2019-05-27

**Authors:** Lea Landolt, Jessica Furriol, Janka Babickova, Lavina Ahmed, Øystein Eikrem, Trude Skogstrand, Andreas Scherer, Salwa Suliman, Sabine Leh, James B. Lorens, Gro Gausdal, Hans‐Peter Marti, Tarig Osman

**Affiliations:** ^1^ Department of Clinical Medicine University of Bergen Bergen Norway; ^2^ Department of Medicine Haukeland University Hospital Bergen Norway; ^3^ BerGenBio ASA Bergen Norway; ^4^ Department of Biomedicine University of Bergen Bergen Norway; ^5^ Spheromics Kontiolahti Finland; ^6^ Institute for Molecular Medicine Finland FIMM HiLIFE University of Helsinki Helsinki Finland; ^7^ Department of Clinical Dentistry Center for Clinical Dental Research University of Bergen Bergen Norway; ^8^ Department of Pathology Haukeland University Hospital Bergen Norway; ^9^ Department of Biomedicine Center for Cancer Biomarkers University of Bergen Bergen Norway

**Keywords:** AXL targeting, Bemcentinib (BGB324), renal fibrosis, UUO

## Abstract

The AXL receptor tyrosine kinase (RTK) is involved in partial epithelial‐to‐mesenchymal transition (EMT) and inflammation – both main promoters of renal fibrosis development. The study aim was to investigate the role of AXL inhibition in kidney fibrosis due to unilateral ureteral obstruction (UUO). Eight weeks old male C57BL/6 mice underwent UUO and were treated with oral AXL inhibitor bemcentinib (*n* = 22), Angiotensin‐converting enzyme inhibitor (ACEI,* n* = 10), ACEI and bemcentinib (*n* = 10) or vehicle alone (*n* = 22). Mice were sacrificed after 7 or 15 days and kidney tissues were analyzed by immunohistochemistry (IHC), western blot, ELISA, Sirius Red (SR) staining, and hydroxyproline (Hyp) quantification. RNA was extracted from frozen kidney tissues and sequenced on an Illumina HiSeq4000 platform. After 15 days the ligated bemcentinib‐treated kidneys showed less fibrosis compared to the ligated vehicle‐treated kidneys in SR analyses and Hyp quantification. Reduced IHC staining for Vimentin (VIM) and alpha smooth muscle actin (*α* SMA), as well as reduced mRNA abundance of key regulators of fibrosis such as transforming growth factor (*Tgfβ*), matrix metalloproteinase 2 (*Mmp2*), *Smad2*,* Smad4*, myofibroblast activation (*Aldh1a2*,* Crlf1*), and EMT (*Snai1,2, Twist*), in ligated bemcentinib‐treated kidneys was compatible with reduced (partial) EMT induction. Furthermore, less F4/80 positive cells, less activity of pathways related to the immune system and lower abundance of MCP1, MCP3, MCP5, and TARC in ligated bemcentinib‐treated kidneys was compatible with reduction in inflammatory infiltrates by bemcentinib treatment. The AXL RTK pathway represents a promising target for pharmacologic therapy of kidney fibrosis.

## Introduction

Chronic kidney diseases (CKD) are on the rise in many parts of the world, representing a global public health issue (Eckardt et al. 2013; USRDS, 2016). The worldwide prevalence of CKD is around 13% for stages 1–5 and 10% for stages 3–5 and is expected to rise due to increased prevalence of obesity, type 2 diabetes mellitus, hypertension, and also longevity (Jha et al. 2013; Mills et al. 2015; Hill et al. 2016). The age standardized death rate for CKD has increased by more than 30% between 1990 and 2013 and this trend is expected to continue (Eckardt et al. 2013; GBD, 2015). High mortality of CKD has been attributed to accelerated vascular calcification and cardiovascular diseases (Go et al. 2004; Stenvinkel 2010). The burden on the health care system increases with CKD severity; foremost end stage renal disease is costly as a result of long‐term dialysis and transplantation (Honeycutt et al. 2013).

Kidney fibrosis is the morphological correlate of progressive CKD and is characterized by the accumulation of myofibroblasts with subsequently substantial extracellular matrix (ECM) deposition, inflammation, tubular epithelial cell loss, and microvascular rarefaction (Kuncio et al. 1991; Zeisberg and Neilson 2010). Activated myofibroblasts are one of the key cells in the pathogenesis of fibrosis, and it was initially suggested that they are derived mainly from tubular epithelial cells through epithelial‐to‐mesenchymal transition (EMT) (Iwano et al. 2002). Recent lineage tracing experiment reported that proliferation of tissue resident fibroblasts, differentiation of bone marrow precursors and endothelial to mesenchymal transition all together form the majority of the myofibroblast pool in kidney fibrosis, while less than 5% are directly derived from the renal tubular cells by EMT (LeBleu et al. 2013). Accordingly, an incomplete, “partial” EMT of the renal tubular epithelial cells has been suggested to promote fibrosis development through cell‐cycle arrest and paracrine secretion of growth factors and cytokines – including TGF‐*β* – that trigger the activation of myofibroblasts and induce inflammation (Ovadya and Krizhanovsky 2015; Lovisa et al. 2016; Nieto et al. 2016). Key transcription factors driving partial EMT of tubular epithelial cells are SNAI1/2, TWIST, and STAT1 (Lee et al. 2014; Grande et al. 2015; Lovisa et al. 2015). In addition, the inflammatory response to kidney injury, although aimed to preserve the integrity of the tissue at the initial stages of CKD, provides a reservoir of cytokines and enzymes that promote the ongoing fibrosis process (Lovisa et al. 2016).

CKD progression can be attenuated in its early phases by control of blood pressure, proteinuria, and therapy addressing the underlying disease, such as diabetes mellitus, hypertension or glomerulonephritis. Due to high morbidity and mortality related to kidney diseases, there is a need for new therapeutic strategies that target the fibrosis‐related mechanisms in the kidney. The transmembrane AXL receptor belongs to the TAM family of receptor tyrosine kinases (RTK) together with MERTK and TYRO3 and is an important mediator of inflammation, as well as EMT in malignancies (Korshunov 2012; Axelrod and Pienta 2014; Feneyrolles et al. 2014; Dransfield and Farnworth 2016). In accordance, AXL RTK inhibition blocks tumorigenicity and reduces aggressiveness of solid tumors (Janning et al. 2015; Yu et al. 2015). AXL RTK is thus an important therapeutic target in cancer and the small molecule bemcentinib, a selective AXL RTK inhibitor (formerly called BGB324), is currently in phase II clinical trials for e.g. AML (NCT02488408), NSCLC (NCT02424617; NCT02922777), melanoma (NCT02872259) and breast cancer (NCT03184558) (ClinicalTrials.gov, 2018).

In solid organ fibrosis, AXL RTK has been studied in experimental liver, lung, and renal fibrosis (Barcena et al. 2015; Espindola et al. 2018; Zhen et al. 2018). However, the effect of AXL RTK inhibition in kidney fibrosis remains to be a relatively understudied area (Batchu et al. 2013; Hyde et al. 2014). Unilateral ureteral obstruction (UUO) represents an established animal model for renal fibrosis development that also involves partial EMT as a main driver (Grande et al. 2015; Lovisa et al. 2015). Within 1 day after obstruction, reduction in renal perfusion occurs, which is followed by hydronephrosis and fibrosis development within a few days, and loss and flattening of the cortex within 1–2 weeks. The contralateral nonligated kidney remains histologically normal and can therefore serve as a control (Vaughan et al. 2004; Chevalier et al. 2009).

The hypothesis of this study was that AXL RTK is involved in fibrosis development in UUO and that inhibition of AXL RTK with the selective small molecule bemcentinib leads to the reduction in fibrosis development in UUO.

## Methods

### Animals

Eight to nine‐week‐old male C57Bl/6JOlaHSD mice were obtained from Envigo (Horst, The Netherlands). All animals were kept in the local animal facility of the Department of Biomedicine at the University of Bergen, Norway. The experiments were conducted in accordance with the guidelines and approval of the Norwegian Food Safety authority (Approval numbers: 16/116548 and 17/129461).

### Unilateral ureteral obstruction

All surgical procedures were performed under isoflurane anaesthesia (Isoflurane, Baxter, Oslo, Norway. ATC‐nr: N01A B06). The operation site was shaved with an electric shaver, disinfected, and the left ureter was identified through a subcostal incision and obstructed using a silk ligature at the level of the lower pole of the kidney. Peritoneum and muscles were sewn with Prolene 3‐0 (Ethicon, polypropylen, Summerville, NJ USA) and the skin was clipped with “ez clip wound closures” (Stoelting, Wood Dale, USA). Mice were treated with buprenorphine (Temgesic, Indivior, ATC‐nr: N02A E01) 0.1 mg/kg after the operation (Tveitaras et al. 2015).

### Experimental setup

**Figure 1:**
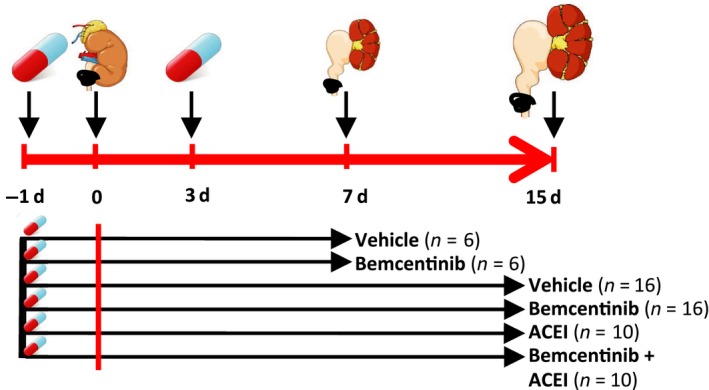
Experiment setup. Experiment setup of unilateral ureteral obstruction (UUO) in male C57Bl/6 mice. Pretreatment of C57Bl/6 mice with either bemcentinib, ACE inhibitor (ACEI), bemcentinib plus ACEI or vehicle was started 1 day before the operation. Mice were subjected to UUO at day 0. Subsequently, mice were sacrificed on day 7 (*n* = 6 for bemcentinib, *n* = 6 for vehicle‐treated mice) or 15 (*n* = 16 for bemcentinib, *n* = 10 for ACEI, and bemcentinib plus ACEI and *n* = 16 for vehicle‐treated mice) after the operation.

The experimental setup and treatment groups are illustrated in Figure 1. Animals were divided in groups treated with bemcentinib, [(1‐(6,7‐dihydro‐5H‐benzo[6,7]cyclohepta[1,2‐c]pyridazin‐3‐yl)‐N3‐((7‐pyrrolidin‐1‐yl)‐6,7,8,9‐tetrahydro‐5H‐benzo[7]annulene‐2‐yl)‐1H‐1,2,4‐triazole‐3,5‐diamine)], its solvent Vehicle (0.5% hydroxylpropyl‐methylcellulose in 0.1% tween 80), Angiotensin‐converting enzyme inhibitor (ACEI, Enalapril maleate salt, Sigma‐Aldrich, E6888) or ACEI plus bemcentinib. Bemcentinib was administered by oral gavage at a dose of 50 mg/kg (10 mL/kg) twice daily beginning 1 day before or 3 days after operation. The ACEI was dissolved in Methanol and supplied in the drinking water at 100 mg/L and a fresh solution was prepared every third day (Moridaira et al. 2003).

### Sacrifice of mice and sample collection

Animals were sacrificed on day 7 or day 15 after the operation. Mice were anesthetized with isoflurane, the abdominal aorta was dissected and cannulated in order to perfuse the organs with ice‐cold PBS (Tveitaras et al. 2015). The kidneys were then removed and cut in transverse slices that were either snap frozen in RNAlater (RNA Stabilization agent; Qiagen, Venlo, the Netherlands, catalog number: AM7020) and stored at −80°C or fixed in 4% formaldehyde to be processed and embedded in paraffin according to standard procedures at the Institute of Pathology, Haukeland University Hospital, Bergen, Norway. Blood was collected using heart puncture at time of sacrifice. Serum creatinine levels were measured by Bevital AS, Bergen, Norway, using established HPLC‐MS/MS methodology (Midttun et al. 2013).

### Immunohistochemistry and Sirius Red staining

FFPE sections (3 *μ*m) from the ligated and the nonligated kidneys were deparaffinized in xylene and rehydrated in decreasing concentrations of ethanol. Sections subjected to Sirius Red (SR) staining were incubated with a solution of 0.1% SR F3B (BDH Laboratory supplies, catalog number: 34149) in saturated aqueous picric acid for 30 min. Sections for immunohistochemistry were subjected to microwave heat (pH 9.0 or pH 6.0 target retrieval buffer, Dako) or enzyme‐induced epitope retrieval as shown in Table 1, followed by 10 min incubation with Peroxidase block (Dako) to quench endogenous peroxidase activity. Blocking of unspecific binding sites was achieved by incubating the section with 10% normal goat serum (Dako) or Avidin/Biotin block (Vectorlabs) depending on the detection method used. Primary antibodies were diluted in antibody diluent with background reducing agent (Dako). Dilutions, incubation times, and retrieval methods employed for each reaction are listed in Table 1. Labeled polymer‐Horseradish peroxidase conjugated to goat antimouse or antirabbit immunoglobulins (Envision+^®^ system, Dako) was used to detect immunoreactivity of anti‐Vimentin (VIM), anti‐alpha Smooth muscle actin (*α*SMA), anti‐N‐cadherin (CDH2) and anti‐Twist antibodies. Tissue bound anti‐AXL antibody was detected using 30 min incubation with rabbit anti‐goat IgG (1:1000, SouthernBiotech), followed by incubation with Envision+^®^ polymer. Anti‐F4/80 antibody was detected using 30 min incubation with biotinylated goat anti‐rat IgG (1:300, Vectorlabs), followed by Vectastain ABC‐HRP reagent (Vectorlabs). All immunoreactions were visualized using 3,3′‐diaminobenzidine (DAB, Dako), counterstained with Hematoxylin (Dako), dehydrated and cover‐slipped using nonaqueous mounting medium. All reagents and kits were used by following the manufacturer`s instructions.

**Table 1:** Antibodies used in immunohistochemistry

Antibody	Company	Catalog number	Epitope retrieval	Dilution	Incubation
Monoclonal rabbit anti‐VIM	Abcam	ab92547	pH 9	1:3000	1 h
Purified rat anti‐ F4/80	BioRad	MCA497GA	Proteinase K	1:300	Overnight
Polyclonal goat anti‐ AXL	R & D systems	AF854	pH 9	1:1000	1 h
Monoclonal mouse anti‐*α*SMA	Dako	M0851	No retrieval	1:200	30 min
Monoclonal mouse anti‐CDH2	Abcam	ab98952	pH 6	1:1000	1 h
Monoclonal mouse anti‐TWIST	LSBio	SAB4504605	pH 6	1:2000	1 h

Antibodies against Vimentin (VIM), F4/80, AXL, alpha Smooth muscle actin (*α*SMA), C‐cadherin (CDH2), and TWIST used for immunohistochemical stainings in this study, including their provenance, mode of epitope retrieval, dilution, and incubation duration.

### Quantification of immunohistochemistry and Sirius Red stainings

Stained slides were scanned in ScanScope™ system (Aperio, Vista, California, USA) with a resolution of 0.23 *μ*m per pixel, and the generated digital slides were viewed in Imagescope 12 (Leica Biosystems, Nussloch, Germany). Quantification of SR and IHC stainings were carried out using the color deconvolution algorithm version 9 (Aperio), and the red, green, and blue (RGB) values for SR or DAB were obtained as described elsewhere (Ruifrok and Johnston 2001; Osman et al. 2013; Rosa et al. 2013). The obtained percentages of positive pixels were compared between different treatment groups.

### Scoring of cortical tissue loss

Central transversal sections of the kidney were stained with PAS. The sections were reviewed by two of the authors (S. L. and J. B.) in a blinded manner. Cortex thickness and pelvis dilatation were semiquantitatively scored according to a visual grading scale from 0 to 4, where 0 corresponds to normal cortex thickness and unremarkable pelvis and 4 corresponds to maximal cortex thinning and pelvis dilatation (O'Neill 2000).

### Quantification of collagen deposition by hydroxyproline measurement

The colorimetric QuickZyme^®^ total collagen assay was utilized for quantification of collagen deposition by hydroxyproline (Hyp) measurement (QuickZyme Biosciences, Leiden, The Netherlands, catalog number: QZBtotcol1). Aliquots of 10–25 mg of snap frozen mouse renal tissues were first hydrolyzed in 6 mol/L HCl for 20 h at 95°C. The lysates were diluted to 4 mol/L HCl and then added in triplicates to a 96‐well plate. Assay buffer and detection reagent were then added and the plate was incubated for 60 min at 60°C and read at 570 nm on a spectrophotometer. Quantity of total proteins was determined by using the QuickZyme total protein assay (QuickZyme, catalog number: QZBtotprot1). Following the addition of assay buffer and a color reagent, the assay plate was incubated at 85°C for 60 min and then read at 570 nm on a spectrophotometer. Read‐outs of the collagen assay were normalized to total protein contents of the tissue samples.

### Western blot

Protein extraction from mouse kidney tissues was achieved by using RIPA buffer (Sigma‐Aldrich, catalog number: R0278) with the addition of complete protease inhibitor (Roche, catalog number: 4693116001) and phosphatase inhibitor cocktail (Sigma‐Aldrich, catalog number: P5726). Protein concentration was determined using Pierce BCA Protein Assay Kit (Thermo Scientific, catalog number: 23225). Proteins were separated in Bolt 4–12% Bis‐Tris Plus electrophoresis gels and transferred to nitrocellulose membranes using iBlot 2 System. Membranes were blocked with 5% BSA in PBS containing 0.1% Tween‐20 and then incubated overnight with goat anti‐mouse AXL (R&D, catalog number: AF854), or with rabbit anti‐SNAIL+SLUG (Abcam, ab180714). SeeBlue Plus2 Pre‐stained Protein Standard (Invitrogen, LC5925) was used to visualize protein molecular weight. The blots were washed three times with a wash buffer (PBS, 0.1% Tween‐20) and then incubated for 1 h either with rabbit anti‐goat (Southern Biotech, catalog number: 6164‐01), or goat anti‐rabbit (Abcam, ab205718) secondary HRP‐linked antibodies for detection of AXL and SNAIL, respectively. The blots were washed again and developed using Pierce ECL Plus Western blotting substrate (Thermo Fisher). Chemo‐luminescence signals were assessed using Syngene G:Box camera and GeneSys software (Cambridge, UK). Densitometry analysis was performed using the ImageJ.

### Quantification of chemokines by multiplex bead immunoassays

Chemokine levels were analyzed using a multiplex fluorescent bead‐based immunoassay (Bio‐Plex Pro Mouse Chemokine Assay, Bio‐Rad Laboratories, catalog number: 12002231 and Y60000CMCZ). The Bio‐Plex 200 System processed by Luminex was used, including microplate platform and Bio‐Plex Manager 6 software. The analysis was done according to the manufacturer's recommendations based on the Luminex xMAP technology. The amount of protein in each sample was extrapolated and compared with the standard curve ranges with concentrations reported in pg/mL (Houser 2012).

### RNA extraction and mRNA sequencing

For RNA extraction from frozen murine tissues, we used the RNeasy mini kit (Qiagen, catalog number: 74104). To determine the quality of extracted RNA, we used the Agilent 2100 BioAnalyzer and RNA 6000 Nano total RNA kit (Agilent Technologies, catalog number: 5067‐1511). Sequencing libraries of murine tissues were prepared using the TruSeq stranded mRNA library preparation kit (Illumina, USA) and sequenced on an Illumina HiSeq4000 platform at the Genomics Core Facility at the Department of Clinical Medicine at Haukeland University Hospital, Bergen, Norway.

### Analysis of mRNA sequencing data

Reads of murine mRNA were aligned to GRCm38, M13 Gencode release using HISAT2 2.0.5 (Kim et al. 2015). Aligned reads present within adequate GENCODE gene annotation regions (Harrow et al. 2006) were counted using featureCounts (Liao et al. 2014). Count data were analyzed using the LIMMA package in R bioconductor ( https://www.bioconductor.org/packages/release/bioc/html/limma.html) (Ritchie et al. 2015). Preprocessing of count data included log2 transformation and scaling to the library size to obtain log2 cpm values (log2 of counts per million). Voom was applied to estimate nonparametrically the mean‐variance trend of the logged read counts and to use this mean‐variance relationship to predict the variance of each log2cpm value (Law et al. 2014). Further model fitting was performed as outlined (Ritchie et al. 2015). Various contrasts were then applied to obtain statistically significant expression changes. An arbitrary cutoff (Benjamini‐Hochberg adjusted *P*‐value of 0.05 and a minimum fold change of 1.5) was applied as criteria for significance. Additional computation and data visualization was performed using the software JMP Genomics (v.8.2, www.sas.com), and Graphpad Prism v. 7 ( www.graphpad.com). The “perturbation score” of murine mRNA expression results was calculated as follows: for each sample, normalized log2cpm values were standardized to the average normalized log2cpm of the nonligated samples. Ratios smaller than 1 were inversed, so that values for under‐ and over representation would contribute equally to the score and not balance each other out. The average ratio was calculated for all genes by sample, resulting in the perturbation score for each sample. The list of “random genes” was generated within JMP Genomics. The list of metzincins and related genes (MARGS) was taken from our previous publication (Marti et al. 2014). The list of macrophage‐related genes were taken from (Mosser and Edwards 2008). All other gene lists were extracted from SABiosciences ( http://gncpro.sabiosciences.com/gncpro/gncpro.php). The gene lists are presented in the Table S1.

The sequencing data are available in the repository Gene Expression Omnibus https://www.ncbi.nlm.nih.gov/geo/query/acc.cgi?acc=GSE123674.

### Gene ontology and pathway analysis

Significantly up‐ and down‐regulated genes were analyzed for enrichment of gene ontology (GO) terms (including biological processes in particular) and KEGG pathway by using PantherDB ( www.pantherdb.org) (Mi et al. 2017). In the results, we mainly focused on “biological processes” because these are the most widely used for GO analysis. False Discovery Rate (FDR) values lower than 0.05 were considered significant.

### Statistics

Results are presented as mean ± standard deviation. Pair‐wise comparisons between the ligated and contralateral nonligated kidneys were performed by paired sample *t*‐test. The unpaired *t*‐test was used to compare differences between two groups, such as mRNA abundance in ligated bemcentinib‐treated or vehicle‐treated kidneys. For multiple group comparisons such as in SR or IHC staining and hydroxyproline content measurement analyses, one‐way ANOVA was performed, always followed by post hoc analysis with Bonferroni correction. Values of *P* ≤ 0.05 were regarded as significant. *P* values were marked with * (≤0.05), ** (≤0.01), *** (≤0.001), and **** (≤0.0001). All analyses were performed in Statistical Package for Social Sciences (SPSS, Version 25, IBM Analytics).

## Results

### Ureteral ligation induces fibrosis, inflammation, and up‐regulation of AXL

**Figure 2:**
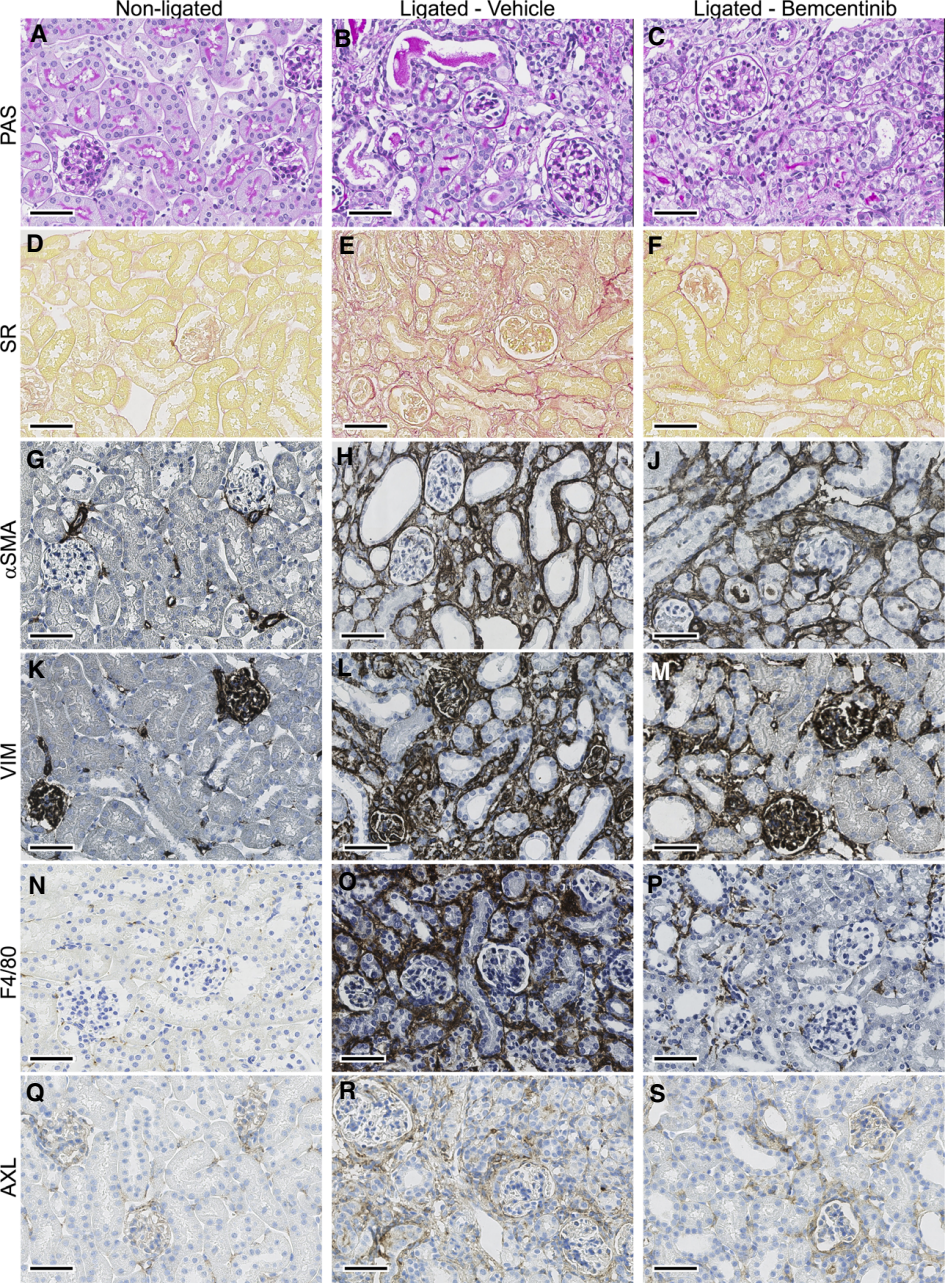
Representative histological images of kidney tissues harvested from male C57Bl/6 mice after 15 days of ureteral obstruction. Formalin fixed paraffin embedded sections from nonligated (left column), ligated vehicle‐treated (middle column) or ligated bemcentinib‐treated kidneys (right column), stained with PAS (A–C), Sirius Red (SR, D–F), or stained by immunohistochemistry (brown) for alpha Smooth muscle actin (*α* SMA, G–J), Vimentin (VIM, K–M), F4/80 (N–P) or AXL (Q–S). Unilateral ureteral obstruction leads to tubulointerstitial fibrosis and tubular atrophy in the ligated kidney compared to the nonligated kidney as seen in the Periodic Acid Schiff staining (PAS, A, B). Counterstaining with hematoxylin. Scale bar is 50 *μ*m.

To characterize the degree of EMT development and AXL expression of the UUO model, we first analyzed the effect of ureteral obstruction in terms of fibrosis, EMT, and inflammation by pairwise comparison of ligated and contralateral nonligated kidneys harvested from the vehicle‐treated group at day 15 (*n* = 16). Histological examination identified visible tubulointerstitial fibrosis and tubular atrophy in the ligated kidneys (Fig. 2A and B). Quantification of the percentage of total positive pixels in SR stained kidney sections (Fig. 2D and E) revealed a significantly higher amount of collagen deposition in the ligated kidneys (mean percentage (%) of positive pixels 14.5 ± 7.4% standard deviation (SD) as compared to the contralateral nonligated kidneys (2.5% ± 2.4%, paired samples *t*‐test, *P*‐value < 0.001). These findings were further supported by colorimetric quantification of Hyp in kidney lysates of the same mice where significantly higher signals were detected from the ligated kidneys (7.76 ± 2.39 *μ*g Hyp/mg total protein) in comparison to the contralateral kidney (2.37 ± 0.59 *μ*g Hyp/mg total protein, *P*‐value < 0.001). Moreover, IHC staining for *α*SMA and VIM (Fig. 2G–L) also showed more staining in ligated compared to nonligated kidneys; an evidence of higher proliferation and activation of myofibroblasts. The mean percentage of positive pixels for VIM was found to be 56.5 ± 5.7% in the ligated kidneys, while contralateral kidneys scored 18.69 ± 1.85% (*P*‐value < 0.001). In the case of *α*SMA, the difference was also found to be statistically significant (*P*‐value = 0.006), with ligated kidneys having 70.13 ± 11.65% compared to 25.2 ± 7.68% for the nonligated kidneys.

**Figure 3:**
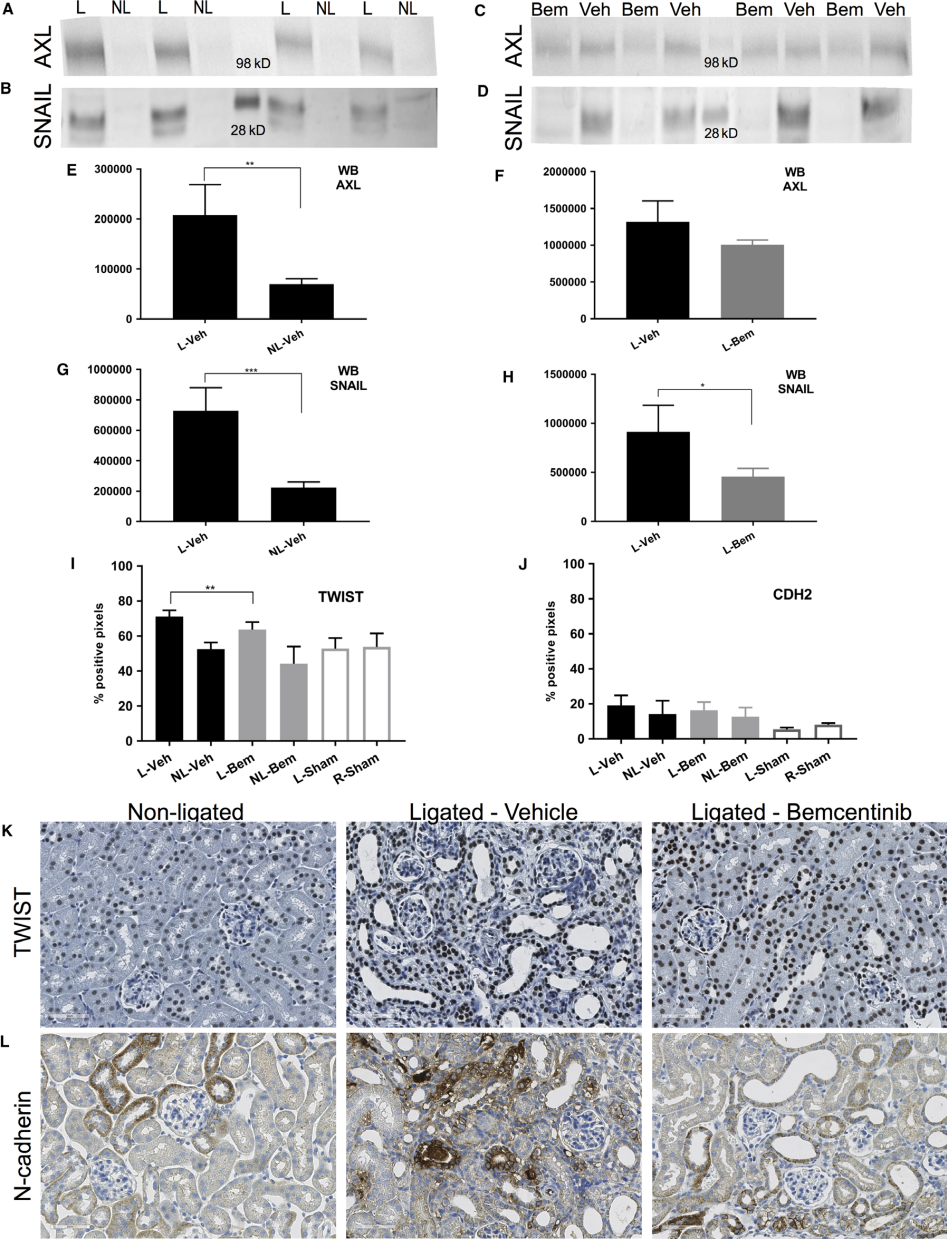
Protein levels of EMT‐related markers in UUO and the effect of Bemcentinib treatment. Comparison of AXL (A) and SNAIL (B) concentrations in ligated (L) and nonligated (NL) kidney lysates from vehicle‐treated mice after 15 days of obstruction. AXL (C) and SNAIL (D) abundance in ligated kidneys from bemcentinib (Bem‐L) and vehicle (Veh‐L)‐treated kidney lysates. Quantification of the WB bands intensity (E–H). Immunohistochmical staining of TWIST (K) and N‐cadherin (L) in nonligated (left), ligated vehicle‐treated (middle) or ligated bemcentinib kidneys (right).

Expression of EMT‐related markers was also found to be increased by ureteral obstruction, and AXL staining was detected in the intertubular interstitial cells, sporadically apical in the tubular epithelial cells of atrophic tubules and in parietal cells of the Bowman′s capsule in glomeruli (Fig. 2Q and R). Additionally, higher amounts of AXL and SNAIL proteins were detected in the lysates of ligated kidneys in comparison to nonligated in western blot (Fig. 3A, B, E and G). Ligated vehicle‐treated kidneys showed higher expression of TWIST (percentage of positive pixels 70.7 ± 4.4%) as compared to the contralateral kidney (52.6 ± 3.8%, *P* = 0.002) by IHC (Fig. 3I and K). No statistically significant difference in CDH2 expression was detected by quantification of IHC (Fig. 3J and L). Taken together, these findings indicate induction of a partial EMT program by kidney ligation as evident by altered expression of SNAIL, TWIST, AXL but not CDH2.

Ligated kidneys were found to be highly infiltrated by F4/80 positive cells (30.8 ± 12.4% positive pixels) as compared to their nonligated counterparts (4.9 ± 1.8%, *P* = 0.008). Additionally, analysis of inflammatory cytokines in kidney tissue lysates by ELISA showed significantly elevated levels of several inflammatory cytokines in the ligated kidneys (Table 2). Moreover, fibroblast growth factor (FGF) was found to be higher in lysates from ligated kidneys (4590 ± 906.5 pg/mL) in comparison to 670 ± 68.4 pg/mL in the contralateral kidneys (Table 2).

**Table 2:** Cytokines, chemokines, and proteins as measured by ELISA in ligated and nonligated kidneys 15 days after obstruction

Chemokine	Kidney	Mean	*n*	SD	*P*‐value
Eotoxin2 (CCL24)	Ligated	2770.4780	5	380.88481	0.016
	Nonligated	1484.1840	5	383.64506	
Fractalkine (CX3CL1)	Ligated	524.9280	5	108.32174	0.029
	Nonligated	309.2780	5	44.58575	
KC (CXCL1)	Ligated	52.4360	5	10.90941	0.003
	Nonligated	32.6000	5	9.16972	
MCP1 (CCL2)	Ligated	460.5320	5	94.53758	0.000
	Nonligated	235.1500	5	46.73821	
MCP3 (CCL7)	Ligated	106.0100	5	13.11334	0.002
	Nonligated	22.8660	5	4.54817	
MIP2 (CXCL2)	Ligated	677.6900	5	66.70261	0.000
	Nonligated	25.9520	5	6.93674	
TARC (CCL17)	Ligated	164.9520	5	35.77862	0.004
	Nonligated	82.7700	5	5.96584	
MCP5 (CCL 12)	Ligated	527.6900	5	170.31400	0.002
	Nonligated	24.7700	5	9.47600	
IL‐8	Ligated	100.1667	6	29.47146	0.000
	Nonligated	670.6667	6	68.40955	
FGF	Ligated	4590.5000	6	906.44928	0.000
	Nonligated	133.3333	6	32.92820	
M‐CSF	Ligated	267.0000	6	31.89984	0.000
	Nonligated	33.6667	6	7.14609	
VEGF	Ligated	33.8333	6	6.99762	0.000
	Nonligated	363.0000	6	52.86965	
IL‐5	Ligated	6.0000	6	1.41421	0.000
	Nonligated	37.8333	6	4.62241	
G‐CSF	Ligated	15.1667	6	4.62241	0.001
	Nonligated	1.8333	6	0.40825	

Differences in the mean protein concentration (pg/mL) were investigated for statistical significance by paired sample *t*‐test.

### Treatment with bemcentinib reduces fibrosis development in obstructed kidneys

**Figure 4:**
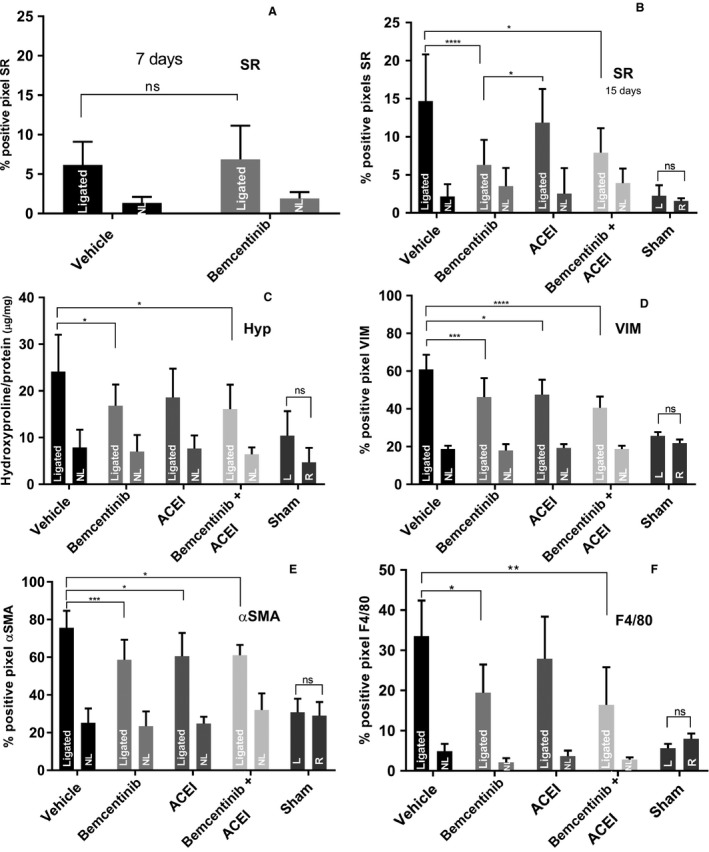
Quantification of Sirius Red staining, immunohistochemistry and hydroxyproline content. Bar charts representing the mean percentage of positive pixels of Sirius Red (SR) staining after 7 days (A) and 15 days (B), the mean percentages of positive pixels of immunohistochemistry for Vimentin (D), *α* SMA (E) and F4/80 (F), and hydroxyproline (Hyp, C) content quantification of ligated (L) and nonligated (NL) kidneys treated with vehicle, bemcentinib, Angiotensin‐converting enzyme inhibitor (ACEI) or bemcentinib + ACEI (B) after 15 days of obstruction. Error bars represent standard deviation.

Differences in fibrosis development between ligated kidneys from bemcentinib and vehicle‐treated mice were not detectable after 7 days of obstruction, and renal tissues only displayed a low grade of fibrosis in SR staining as measured by Aperio (6.2 ± 2.9% positive pixels in ligated bemcentinib‐treated and 6.9 ± 4.3% in ligated vehicle‐treated kidneys, *P* = 0.74) (Fig. 4A). After 15 days, the amount of fibrotic tissue as measured by SR staining remained low in the ligated bemcentinib‐treated kidneys (mean 6.3 ± 3.3%), but was significantly increased in the ligated vehicle‐treated group (14.7 ± 6.1% SD, *P* < 0.001, one‐way ANOVA) (Figs. 2E, F and 4B). Treatment with ACEI did not result in a significant reduction in fibrosis development in the ligated kidneys compared to the ligated vehicle‐treated controls (11.9 ± 4.4% in ligated ACEI‐treated kidneys). However, bemcentinib treatment resulted in less fibrosis development as measured by SR staining in ligated kidneys as compared to ligated ACEI‐treated kidneys (*P* = 0.026, one‐way ANOVA). Combined treatment with bemcentinib plus ACEI did not show an additive effect on fibrosis development in ligated kidneys compared to bemcentinib treatment alone (7.9 ± 3.2% in ligated bemcentinib plus ACEI‐treated kidneys) (Fig. 4B).

In accordance with SR staining, significantly less Hyp content was measured in the ligated bemcentinib‐treated compared to the ligated vehicle‐treated kidneys after 15 days (16.8 *μ*g Hyp per mg total protein (*μ*g/mg) ± 4.6 *μ*g/mg SD in ligated bemcentinib‐treated kidneys and 24.2 ± 7.9 *μ*g/mg in ligated vehicle‐treated kidneys, *P* = 0.014, one‐way ANOVA). Bemcentinib showed a tendency to less fibrosis development in ligated kidneys compared to the ACEI‐treated group as measured by Hyp quantification, but the difference was not statistically significant. However, ACEI had no significant effect on fibrosis development in ligated kidneys compared to vehicle treatment (18.6 ± 6.2 *μ*g/mg in the ligated ACEI‐treated kidneys, *P* = 0.291 in one‐way). Again, bemcentinib plus ACEI did not have an additive effect (Fig. 4C). Sham operated mice did not show any sign of fibrosis or other renal damage.

Semiquantitative scoring of the cortex thickness and pelvis dilatation in ligated kidneys, as described in Methods, showed a lower score for bemcentinib‐treated ligated kidneys (Score 1.7 ± 0.8 SD) as compared to vehicle treatment (Score 2.8 ± 1.2 SD, *n* = 16, *P* = 0.015). This corresponds to preservation of more cortical tissue as a result of bemcentinib therapy.

Taken together, these findings confirm less fibrosis development through inhibition of AXL RTK.

### Attenuated EMT and myofibroblast activation in ligated kidneys by bemcentinib treatment

After 15 days of obstruction, percentage of positive pixels for IHC of VIM was significantly lower in the ligated bemcentinib‐treated compared to the ligated vehicle‐treated kidneys (46.3 ± 10.0% in ligated bemcentinib‐treated, 61.0 ± 7.8% in ligated vehicle‐treated kidneys, *P* = 0.0002, one‐way ANOVA). Percentage of positive pixels for IHC of *α*SMA was significantly lower in the ligated bemcentinib‐treated compared to the ligated vehicle‐treated kidneys (58.7 ± 10.6% SD in ligated bemcentinib‐treated, 75.7 ± 9.0% in ligated vehicle‐treated kidneys, *P* = 0.00016, one‐way ANOVA) (Figs. 2G, M and 4D, E). Bemcentinib treatment lead to significantly less VIM and *α*SMA staining in ligated kidneys compared to the ligated vehicle‐treated controls, but combined treatment with ACEI did not have an additive effect (Fig. 4D and E).

Statistically significant difference between bemcentinib‐ and vehicle‐treated ligated kidneys was detected by quantification of IHC TWIST (Bemcentinib 63.7 ± 4.3%, 71.1 ± 3.6% Vehicle, *P* = 0.003, Fig. 3I and K). On the other hand, bemcentinib treatment did not have a significant effect in the expression of CDH2 as measured by IHC quantification (Fig. 3J and L).

Analysis of mRNA sequencing data showed that bemcentinib treatment resulted in down‐regulation of mRNA abundances of several key genes involved in fibrosis and EMT development in the ligated kidneys as compared to the vehicle‐treated group. A selection of particularly relevant genes is provided in Table 3A. Notably, the key EMT inducing transcription factors *Snai1/2*,* Twist,* and *Stat1* as well as collagens (e.g. collagen III and IV chains), *Tgfβ1*, including *Tgfβr1/2*,* Akt3*,* Elastin,* and *Smad2/4*, were down‐regulated as a consequence of bemcentinib treatment. Accordingly, therapeutic intervention also led to a reduction in *Axl* expression on the mRNA level (Table 3A). The lower abundance of SNAIL was confirmed on the protein level in quantified western blot bands (*P* = 0.002, Fig. 3H). AXL showed a tendency to lower protein abundance in this analysis but with borderline statistical significance (Fig. 3F). Noteworthy, no significant down‐regulation of CDH2 by bemcentinib treatment was detected on the mRNA level (Table 3A). Expression of key myofibroblast‐associated genes (summarized in Table 3B) such as *Aldh1a2* and *Crlf1* were also found to be down‐regulated as an effect of bemcentinib treatment supporting a decreased activation of these cells (Grgic et al. 2014). These findings are compatible with attenuation of fibrosis progression through inhibition of EMT development and fibroblast activation following bemcentinib treatment.

**Table 3:** Gene expression changes in ligated bemcentinib‐treated compared to ligated vehicle‐treated kidneys after 15 days. (A) Fibrosis and EMT‐related genes, and (B) Myofibroblast associated genes (Grgic et al. 2014)

ENSEMBL ID	Symbol	Fold change	AveExpr	*P*.Value	adj.*P*.Val
(A)					
ENSMUSG00000024563	SMAD2	−1.12	5.56	0.019	0.044
ENSMUSG00000024515	SMAD4	−1.13	6.13	0.006	0.017
ENSMUSG00000017466	TIMP2	−1.17	7.82	0.040	0.079
ENSMUSG00000032440	TGFBR2	−1.19	7.99	0.001	0.003
ENSMUSG00000031273	COL4A6	−1.20	3.02	0.013	0.033
ENSMUSG00000002603	TGFB1	−1.24	5.49	0.007	0.018
ENSMUSG00000016356	COL20A1	−1.25	2.07	0.016	0.037
ENSMUSG00000042821	SNAI1	−1.26	2.53	0.017	0.039
ENSMUSG00000028163	NFKB1	−1.27	6.41	0.000	0.000
ENSMUSG00000000303	CDH1	1.29	8.02	0.001	0.10
ENSMUSG00000019699	AKT3	−1.32	5.14	0.000	0.000
ENSMUSG00000022676	SNAI2	−1.33	2.98	0.006	0.016
ENSMUSG00000026104	STAT1	−1.33	5.12	0.031	0.064
ENSMUSG00000002602	AXL	−1.38	7.31	0.000	0.000
ENSMUSG00000026837	COL5A1	−1.40	6.37	0.015	0.036
ENSMUSG00000048126	COL6A3	−1.41	6.71	0.018	0.042
ENSMUSG00000007613	TGFBR1	−1.42	7.06	0.000	0.000
ENSMUSG00000040033	STAT2	−1.42	5.83	0.000	0.000
ENSMUSG00000026042	COL5A2	−1.44	7.25	0.005	0.015
ENSMUSG00000035799	TWIST1	−1.50	0.72	0.001	0.005
ENSMUSG00000029675	ELN	−1.51	5.72	0.018	0.042
ENSMUSG00000025650	COL7A1	−1.57	4.03	0.000	0.001
ENSMUSG00000032332	COL12A1	−1.59	7.57	0.000	0.001
ENSMUSG00000031740	MMP2	−1.60	5.97	0.000	0.001
ENSMUSG00000001119	COL6A1	−1.60	6.43	0.004	0.013
ENSMUSG00000056174	COL8A2	−1.60	2.33	0.005	0.016
ENSMUSG00000026043	COL3A1	−1.60	9.07	0.022	0.048
ENSMUSG00000020241	COL6A2	−1.61	5.96	0.006	0.018
ENSMUSG00000019899	LAMA2	−1.63	6.05	0.000	0.000
ENSMUSG00000024304	CDH2	1.65	3.41	0.04	0.23
ENSMUSG00000028339	COL15A1	−1.71	7.56	0.000	0.000
ENSMUSG00000049723	MMP12	−1.87	3.84	0.001	0.005
ENSMUSG00000039462	COL10A1	−3.92	−0.24	0.001	0.002
(B)					
ENSMUSG00000013584	ALDH1A2	−1.27	5.62	0.001	0.002
ENSMUSG00000007888	CRLF1	−1.34	3.46	0.009	0.023
ENSMUSG00000046179	E2F8	−1.43	1.37	0.022	0.048
ENSMUSG00000033060	LMO7	1.21	6.62	0.002	0.008
ENSMUSG00000063450	SYNE2	−1.25	8.79	0.021	0.046
ENSMUSG00000032232	CGNL1	1.28	8.79	0.000	0.000
ENSMUSG00000034427	MYO15B	1.27	6.78	0.010	0.026
ENSMUSG00000022708	ZBTB20	−1.24	8.27	0.029	0.061

### Fibrosis‐related genes are differentially expressed following bemcentinib treatment

**Figure 5:**
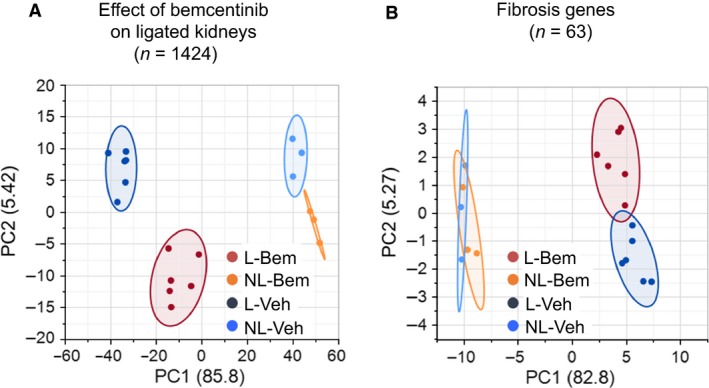
Effect of bemcentinib treatment on gene expression after 15 days of ureteral obstruction. Principal component analysis (PCA) was performed on expression data of genes with statistically relevant difference in expression pattern in ligated kidneys after bemcentinib or vehicle treatment (A), and fibrosis‐related genes (B). In both graphs, the influence of ligation is the largest contributing factor (PC1, in %), followed by the effect of treatment (PC2, in %). In (A), the distance of the group mean centers of ligated and nonligated samples in PC1 is reduced from 76.5 for the vehicle‐treated groups to 59.7 after the bemcentinib treatment, suggesting a reduction of the ligation effect due to bemcentinib treatment. As visual aid, shaded density ellipses outline the probability of 90% of data laying within the contour. L = ligated, NL = nonligated, Bem = bemcentinib‐treated, and Veh = vehicle‐treated.

We evaluated and compared the effect of bemcentinib treatment over 15 days on gene expression in ligated kidneys, as reflected by mRNA abundances. A total of 1424 genes were significantly differentially expressed in bemcentinib‐treated animals compared to vehicle‐treated animals (*n* = 6 for each group) (Fig. 5A). Principal component analysis (PCA) revealed that the ligation effect is the dominant factor explaining the variance of the data (principal component PC1), and the treatment effect appears to be explained by the data variance in PC2). Interestingly, the difference of the group mean of the ligated and nonligated bemcentinib‐treated kidneys is smaller than the corresponding mean difference of the vehicle‐treated kidneys, suggesting a reduction in the ligation effect due to bemcentinib treatment.

To further test the effect of ligation and treatment on gene expression in the tissue, the expression values of 63 fibrosis‐related genes were visualized in a PCA (Fig. 5B). Besides the strong ligation effect (PC1), PC2 reveals that the nonligated samples have a rather large variance but do not segregate into the two treatment groups. The ligated samples however have tendency of forming two clusters according to their treatment group. This indicates that the two groups separate from each other based on the expression pattern of these fibrosis genes. Six genes of this group were also present in the list of 1424 differentially affected genes, such as Angiotensinogen (AGT), Bone morphogenetic protein 7 (BMP7), Chemokine (C‐C motif) ligand 12 (CCL12), Collagen type III, alpha 1 (COL3A1), Matrix metalloproteinase 8 (MMP8), and Plasminogen activator urokinase (PLAU).

### Lower range of expression changes of genes related to EMT, fibrosis, extracellular matrix, MARGS and macrophage markers following bemcentinib treatment

**Figure 6:**
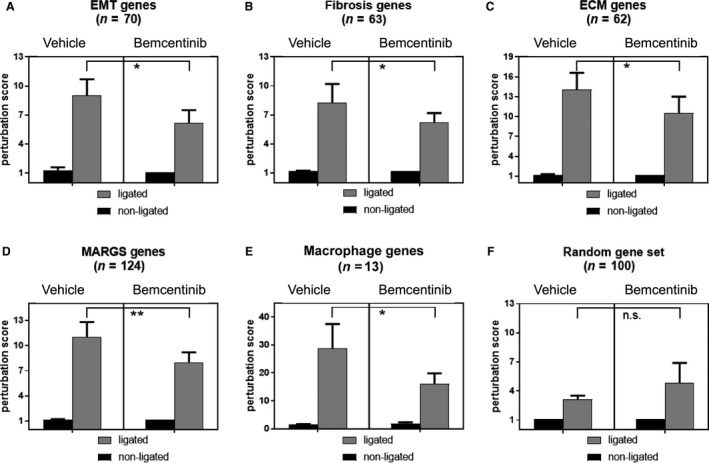
Perturbation scores indicate reduction of ligation effect by bemcentinib treatment after 15 days of ureteral obstruction. Perturbation scores as measures of extent of gene expression changes for six gene lists are presented. (A–F), decrease of perturbation scores in ligated bemcentinib‐treated kidneys applying one EMT (A), three fibrosis (B–D)related gene lists and one gene list with macrophage markers (E) indicates an ameliorating effect of the treatment with bemcentinib on fibrosis development and inflammation in the context of macrophages. (F) A random gene list serves as control for the analysis; its perturbation score difference remains statistically insignificant.

Five gene lists for EMT, fibrosis, extracellular matrix (ECM), metzincins and related genes (MARGS) and macrophage markers were extracted from the literature and publicly available databases as described in Material and Methods (Table S1). A randomly generated gene list served as control. For EMT, fibrosis, ECM, macrophage markers and MARGS, there was significantly less alteration (“perturbation”) of gene expression in the bemcentinib‐treated group compared to the vehicle‐treated group, indicating that expression levels of genes involved in these pathways were less changed. Results are summarized in Figure 6.

### Bemcentinib reduces F4/80+cell infiltration and C‐C motif Chemokine ligands in ligated kidneys

In accordance with less perturbation of macrophage markers in the bemcentinib group (Fig. 6), significantly less F4/80 positive cells were stained in IHC in ligated bemcentinib‐treated compared to ligated vehicle‐treated kidneys after 15 days (19.5 ± 6.9% in ligated bemcentinib‐treated, 33.5 ± 8.9% in ligated vehicle‐treated kidneys, *P* = 0.01, one‐way ANOVA) (Fig. 2N–P). Combined treatment of bemcentinib plus ACEI also yielded significantly less F4/80 positive cells in IHC but again without additive effect compared to bemcentinib alone (16.4 ± 3.4% in ligated bemcentinib plus ACEI‐treated kidneys, *P* = 0.001, one‐way ANOVA). ACEI treatment alone did not alter F4/80 expression (Fig. 4F).

**Figure 7:**
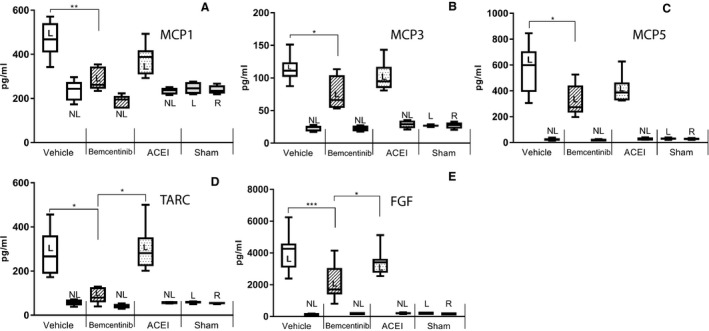
Chemokines and FGF in ligated and nonligated kidneys after 15 days of ureteral obstruction in the presence or absence of bemcentinib treatment. Protein abundance of MCP1 (CCL2) (A), MCP3 (CCL7) (B), MCP5 (CCL12) (C) and TARC (CCL17) (D) and FGF (E) as measured by ELISA in ligated (L) and nonligated (NL) kidneys treated with vehicle, bemcentinib, angiotensin‐converting enzyme inhibitor (ACEI) or bemcentinib + ACEI. Error bars represent standard deviation.

In a multiplex ELISA essay, treatment with bemcentinib was found to reverse several cytokines that were induced by the ureteral ligation. MCP1 (CCL2), MCP3 (CCL7), MCP5 (CCL12), and TARC (CCL17) were significantly less abundant in the lysates from ligated bemcentinib‐treated compared to ligated vehicle‐treated kidneys (both *n* = 5, MCP1 *P* = 0.001, MCP3 *P* = 0.034, MCP5 *P* = 0.028, TARC *P* = 0.008 and FGF *P* < 0.001, one‐way ANOVA). TARC (CCL17) was also significantly underrepresented in ligated bemcentinib‐treated compared to ligated ACEI‐treated kidneys (TARC *P* = 0.004, one‐way ANOVA). These results reflect an anti‐inflammatory effect in the ligated kidneys by bemcentinib treatment compared to the vehicle‐treated controls – probably in particular in the context of monocyte and macrophage‐related infiltrates (Fig. 7).

### Bemcentinib treatment affects metabolic pathways related to immune system and cytokine secretion

**Figure 8:**
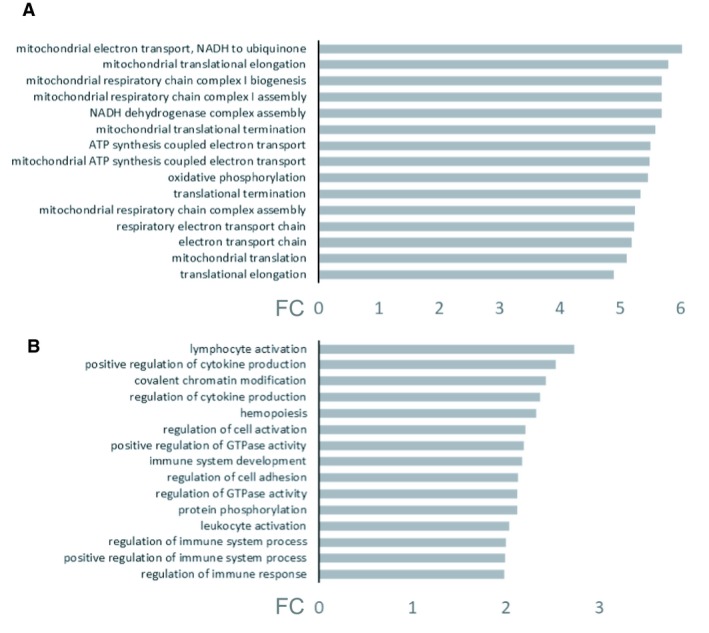
Gene ontology pathway analysis of murine mRNA from ligated bemcentinib and vehicle‐treated kidneys after 15 days of ureteral obstruction. Top 15 up‐ (A) and down‐ (B) regulated gene ontology pathways in ligated bemcentinib‐treated kidneys compared to the ligated vehicle‐treated controls. FC = fold change. Treatment with bemcentinib leads to the up‐regulation of pathways related to mitochondria (such as mitochondrial electron transport, mitochondrial respiratory chain complex I assembly and biogenesis and inner mitochondrial membrane organization as well as mitochondrial ATP synthesis coupled proton transport) (A) and down‐regulation of pathways related to the immune system (lymphocyte and leukocyte activation, cytokine production, immune system development and regulation of immune system processes and response) (B).

Next, genome‐wide transcriptional changes in UUO and the impact of bemcentinib treatment were analyzed. First, we performed transcriptome analysis of ligated compared to nonligated vehicle‐treated kidneys. Gene ontology analysis indicated differences in metabolic pathways mainly related to mitochondria, which were down‐regulated in the ligated kidneys compared to the nonligated kidneys, whereas metabolic pathways associated with EMT and cytokine induction were up‐regulated in the ligated kidneys compared to the nonligated controls (data not shown). Furthermore, the gene ontology analysis comparison of the ligated bemcentinib‐treated compared to ligated vehicle‐treated kidneys, highlights that bemcentinib affects metabolic pathways related to the immune system and cytokine secretion, whose expression was less in ligated bemcentinib‐treated kidneys, and mitochondria‐related pathways, whose expression was higher in ligated bemcentinib‐treated kidneys than in ligated vehicle‐treated kidneys (Fig. 8).

### Renal function remains stable

Renal function was assessed by serum creatinine levels. As expected in UUO, creatinine levels in the serum were not significantly different in the vehicle‐treated UUO mice (16.4 ± 4.7 *μ*mol/L SD; *n* = 12) compared to the bemcentinib‐treated animals (17.9 ± 4.0 *μ*mol/L SD, *n* = 12). These values are only slightly above normal values in mice measured by HPLC (7–13 *μ*mol/L) (Dunn et al. 2004; Palm and Lundblad 2005).

## Discussion

In this study, we have analyzed the potential role of AXL RTK in the progression of fibrosis in the setting of experimental UUO in mice. The underlying study hypothesis was that AXL RTK promotes renal fibrosis development. Accordingly, AXL inhibition should have beneficial effects on the renal architecture with less renal fibrosis development. As a proof of concept, we have therefore subjected male C57Bl/6 mice to UUO for fibrosis induction, which was accompanied by exposure to either bemcentinib, to ACEI as an established clinically used anti‐fibrotic approach, to bemcentinib plus ACEI, or to its vehicle alone as a control during 7 or 15 days. To our knowledge, this is the first study analyzing the effect of AXL RTK inhibition by bemcentinib in UUO.

The UUO model is a well‐established model of progressive renal fibrosis with rapid, consistent disease development allowing sacrifice of animals and analysis after 1 or 2 weeks (Nagle et al. 1973; Chevalier et al. 2009). Moreover, complete ureteral obstruction is a simple procedure with high reproducibility (Yang et al. 2010). The extent of fibrosis after UUO is known to be dependent of the strain and gender of adult mice. In these experiment, we wanted to investigate the anti‐fibrotic effect of bemcentinib and have therefore chosen male C57Bl/6 mice, that are known to develop fibrosis. Female mice and the BALB/c strain are less prone to develop renal fibrosis following UUO (Puri et al. 2010; Cho et al. 2012; Hewitson et al. 2016).

Furthermore, two recent studies have shown that selective depletion of SNAI1 and TWIST inhibits partial EMT development, fibrosis generation and lowers TGF*β* production in ligated kidneys after UUO in mice. Partial EMT thus represents a key feature in UUO‐induced fibrosis (Grande et al. 2015; Lovisa et al. 2015). In our analyses, pair‐wise comparison between ligated and nonligated kidneys showed substantial increase in fibrosis, inflammation, and up‐regulation of several EMT‐related markers including AXL. Taken together, UUO represents an appropriate fibrosis model to investigate the role of the AXL RTK pathway and AXL RTK inhibition in the kidney.

The main endpoint of our study is fibrosis development. There are different methods to quantify organ fibrosis. Accumulation of collagen I is characteristic for fibrosis development and is induced by TGF*β* (Zeisberg et al. 2001; Zeisberg and Neilson 2010). Using SR staining with conventional light microscopy, collagens I, III, and IV are all stained red (Farris and Alpers 2014). *α*SMA is present in activated myofibroblasts contributing to renal fibrosis, accordingly, *α*SMA is a recognized marker of renal fibrosis (Alpers et al. 1993; Chevalier et al. 2009; Zeisberg and Neilson 2010; LeBleu et al. 2013). Another method that reflects fibrosis development is to measure Hyp, a nonproteinogenic amino acid, which especially occurs in collagens, where it stabilizes its triple helix (Berg and Prockop 1973). Hyp content of tissues thus correspond to the total collagen content and this method is used in different settings to quantify collagen deposition in the context of fibrosis development, such as in the UUO model (Sato et al. 2003; Lovisa et al. 2015). Therefore, our analyses support reduced fibrosis development by bemcentinib treatment after 15 days of obstruction, as reflected by decreased staining intensities for SR staining, as well as lower Hyp content. These results are also in line with the respective mRNA sequencing data of fibrosis‐related gene expression. Furthermore, reduced mRNA abundances of individual genes relevant for pathogenesis of fibrosis were observed in the ligated bemcentinib‐treated group, including *Tgfβ1*,* Tgfβ1r1/2*,* Akt3*,* Elastin*,* Laminin* and *Smad2/4* (Yang et al. 2010; Pellicoro et al. 2012; Genovese et al. 2014; Wang et al. 2015).

Further work has to be done to explain the observation that CDH2 protein and mRNA levels were not significantly altered by UUO or bemcentinib treatment. Nevertheless, it has been suggested that CDH1 and CDH2 are regulated in a time‐dependent manner during tubular epithelial injury, and that their use as consistent EMT markers during UUO is questionable (Yang and Liu 2001; Docherty et al. 2009; Grande et al. 2015; Choi et al. 2016).

In SR staining but not in Hyp content measurement, bemcentinib treatment leads to less fibrosis development in ligated kidneys when compared to ACEI. Bemcentinib plus ACEI, however, did not have an additive effect. Previous data showed that rats subjected to UUO for 5 days and treated with ACEI displayed 52% reduction in fibrosis development as compared to the untreated control (Moridaira et al. 2003). The difference in ACEI effect might be due to the fact that our model goes beyond the first 5 days of UUO, which has been reported to be the inflammatory phase of the fibrosis process (Kuncio et al. 1991; Chevalier et al. 2009).

AXL RTK activation is reported to regulate different cellular processes, including survival, proliferation, migration, invasion, angiogenesis, but also processes related to the immune system, where AXL RTK dampens inflammation through different mechanisms such as reduced Toll‐like receptor‐dependent inflammatory signaling and reduced activation of natural killer cells (Graham et al. 2014; Gay et al. 2017). AXL RTK and its sole ligand, GAS6, are overexpressed and activated in many human cancers and correlate with increased invasiveness and metastasis, EMT and drug resistance. Expression of AXL is activated by EMT transcription factors, including SNAI1/2 and AXL RTK activation itself promotes EMT via TWIST and SNAI1/2 (Graham et al. 2014; Akalu et al. 2017; Gay et al. 2017). AXL RTK inhibition reverses EMT in cancer cells and blocks acquired drug resistance to RTK inhibitors (e.g. Sunitinib) and chemotherapy (Korshunov 2012; Yu et al. 2015; Zhou et al. 2016). Based on these findings, it is likely that the AXL RTK pathway plays also a role in the pathogenesis of organ fibrosis and we speculate that attenuated induction of (partial) EMT is one mechanism that explains the reduced fibrosis development by AXL RTK inhibition in UUO. This is reflected by reduced staining for VIM and less activated *α*SMA‐positive myofibroblasts in ligated bemcentinib‐treated compared to ligated vehicle‐treated kidneys and is further supported by down‐regulation of EMT‐related genes, such as *Snai1/2*,* Twist*,* Stat1*,* Zeb1/2*,* Akt3* and *Axl*, and also *Tgfβ* and *Smad2/4*. The latter genes are involved in the canonical pathway of TGF*β*, which is known to be one of the main triggers of EMT (Lamouille et al. 2014; Nieto et al. 2016). The perturbation score, a method to display gene expression differences of multiple genes simultaneously, also supports smaller extent of expression changes of EMT‐related genes following bemcentinib treatment.

Emerging literature support a role for AXL in fibrosis development and an antifibrotic and anti‐inflammatory role of the AXL inhibitor bemcentinib. A study on liver fibrosis showed that AXL and GAS6 are required to induce fibrogenesis by hepatic stellate cells; Accordingly, exposition to bemcentinib reduced liver fibrosis in mice. In addition, less recruitment of antigen presenting cells, particularly macrophages, were detected as measured by F4/80. Less inflammation was also demonstrated by lower expression of chemokines (Barcena et al. 2015). AXL has also been seen to be up‐regulated in idiopathic lung fibrosis and bemcentinib lead to a reduction in fibrosis development in two mouse lung fibrosis models (Espindola et al. 2018). Recently, a study using murine anti–GBM‐induced renal fibrosis models, showed less inflammation with reduced expression of cytokines and chemokines, and an improved renal function upon bemcentinib treatment. Accordingly, a similar effect was found in AXL knock out mice (Zhen et al. 2018).

The UUO model – compared to the anti–GBM‐induced renal fibrosis model in (Zhen et al. 2018) – is not based on autoimmune and inflammatory mechanisms. However, obstruction leads to interstitial inflammation, predominantly by monocyte/macrophage infiltration, already after 3 days and inflammation is seen as an important component and driver of fibrosis development in UUO (Chevalier et al. 2009; Lopez‐Novoa and Nieto 2009).

The observed reduction in inflammation following bemcentinib treatment could possibly also contribute to the attenuation of fibrosis development by bemcentinib in UUO. This was reflected by markedly less F4/80 positive cells and less activated myofibroblasts in ligated bemcentinib‐treated kidneys compared to the ligated vehicle‐treated control. F4/80 is known to stain mostly macrophages, which are important producers of TGF*β* (Docherty et al. 2006; Dos Anjos Cassado 2017). The lower expression of chemokines, cytokines, and the lower degree of expression changes of macrophage marker genes in the perturbation score in ligated bemcentinib‐treated tissues indicate reduced inflammation in ligated kidneys after bemcentinib treatment. Down‐regulation of metabolic pathways related to immune system and cytokine secretion in the ligated bemcentinib‐treated kidneys further support these findings.

Reasons why targeting AXL seems to reduce inflammation in our experiments (and also in experiments performed in liver fibrosis (Barcena et al. 2015)), might be reduced activation of myofibroblasts, which are proinflammatory cells, or also the sustained effects of MERTK, namely efferocytosis and reduced T‐cell activation. MERTK is not affected by bemcentinib due to the high selectivity of the compound to AXL as compared to MERTK (Graham et al. 2014).

It remains to be further investigated, to which degree the attenuated fibrosis development is a consequence of the inhibition of (partial) EMT in renal tubular cells and of inflammation, or a result of other mechanisms such as direct effect on activated renal myofibroblasts.

In another experiment, we initiated treatment with bemcentinib on day 3 after ureteral ligation. Interestingly, delaying bemcentinib treatment did not show reduced fibrosis development after 15 days (data not shown). Reasons for this outcome remain unclear and it requires further investigation if, for example, a missing attenuation of inflammation by bemcentinib within the first 3 days after ureteral obstruction might play a role.

Taken together, the blockade of the AXL RTK pathway reduces renal fibrosis development in the context of UUO. Most likely the mechanisms are a decrease of (partial) EMT induction and reduction in inflammatory response. Therefore, the AXL RTK pathway could be relevant for fibrosis development and progression in many different forms of CKD such as diabetic nephropathy.

In the clinical context, CKD is defined as a reduced renal function measured by glomerular filtration rate, which is often accompanied by proteinuria. A limitation of our study is that UUO is a unilateral model of tubulointerstitial fibrosis without renal function as an end point. Therefore, further experiments should employ renal fibrosis models that enable functional read‐outs to investigate whether inhibition of fibrosis progression by bemcentinib treatment leads to improved glomerular filtration rate and reduced proteinuria. Furthermore and in the future, a suitable AXL knockout model and also proteomic analyses could be performed to supplement our findings in the context of fibrosis development and inflammation alteration by AXL and AXL RTK inhibition.

## Conclusion

The AXL RTK pathway is involved in the pathogenesis of renal fibrosis in UUO and represents a novel and promising target for the pharmacologic prevention of fibrosis development in CKD.

## Conflict of Interest

This study has been supported by BerGenBio ASA, which has also provided the bemcentinib compound. J. B. Lorens declares ownership in BerGenBio ASA. G. Gausdal and L. Ahmed are employees of BerGenBio ASA. Lea Landolt has received a PhD scholarship from the University of Bergen, Norway.

## Supporting information




**Table S1**. Gene lists of gene lists with epithelial‐to‐mesenchymal transition (EMT), fibrosis and extracellular matrix (ECM) related genes, metzincins, and related genes (MARGS), macrophage markers (MPh) and a list of random genes as a control.
