# Supplementary material for: AXL targeting reduces fibrosis development in experimental unilateral ureteral obstruction
Source: Physiol Rep. 2019 May 27;7(10):e14091. doi: 10.14814/phy2.14091 (PMC6536582; doi:10.14814/phy2.14091)
Supplement: Supplementary file 1 — Table S1. Gene lists of gene lists with epithelial‐to‐mesenchymal transition (EMT), fibrosis and extracellular matrix (ECM) related genes, metzincins, and related genes (MARGS), macrophage markers (MPh) and a list of random genes as a control. [file PHY2-7-e14091-s001.docx]

| **EMT**  **(n=70)** | **Fibrosis (n=63)** | **ECM**  **(n=62)** | **MARGS (n=124)** | ***cont.*** | **MPh**  **(n=13)** | **Random (n=100)** | ***cont.*** |
| --- | --- | --- | --- | --- | --- | --- | --- |
| AHNAK | AGT | ADAMTS1 | ADAM10 | MMP15 | SPHK1 | 2610307P16RIK | NFATC1 |
| AKT1 | AKT1 | ADAMTS2 | ADAM11 | MMP17 | RETNLA | 4930581F22RIK | NOP9 |
| BMP1 | BCL2 | ADAMTS5 | ADAM15 | MMP19 | F13A1 | AFAP1L1 | NUAK2 |
| BMP7 | BMP7 | CD44 | ADAM17 | MMP2 | STAB1 | AGXT2 | NUP43 |
| CALD1 | CAV1 | CDH1 | ADAM19 | MMP24 | CXCL9 | ALDH2 | PACS1 |
| CAMK2N1 | CCL11 | CDH2 | ADAM23 | MMP25 | CCL22 | ALKBH8 | PICK1 |
| CAV2 | CCL12 | CDH3 | ADAM33 | MMP28 | CXCL10 | ANXA8 | PLEKHO2 |
| CDH1 | CCL3 | CDH4 | ADAM8 | MMP3 | CCL17 | AREL1 | PRPF40B |
| CDH2 | CEBPB | CNTN1 | ADAM9 | MMP7 | IL27RA | ARHGAP44 | RBM26 |
| COL1A2 | COL1A2 | COL1A1 | ADAMTS1 | MMP8 | NOS2 | ARHGEF4 | REG3G |
| COL3A1 | COL3A1 | COL3A1 | ADAMTS12 | MMP9 | CCL20 | BMF | RIC1 |
| COL5A2 | CTGF | COL4A1 | ADAMTS14 | NFKB1 | IGF1 | BSDC1 | RPLP0 |
| CTNNB1 | CXCR4 | COL4A2 | ADAMTS15 | NFKB2 | CLEC4A2 | BSND | RTKN |
| DSC2 | DCN | COL4A3 | ADAMTS16 | PAPLN |  | C8G | SEPSECS |
| DSP | EDN1 | COL5A1 | ADAMTS17 | PLAT |  | CALHM2 | SMPD1 |
| EGFR | EGF | COL6A1 | ADAMTS2 | PLAU |  | CANT1 | SOS1 |
| ERBB3 | GREM1 | COL8A1 | ADAMTS4 | PRG4 |  | CAPZA2 | STK3 |
| ESR1 | HGF | CTGF | ADAMTS5 | SERPINE1 |  | CAR13 | SULT1B1 |
| F11R | IL1B | CTNNA2 | ADAMTS6 | SERPINE2 |  | CKB | SUSD4 |
| FGFBP1 | ILK | CTNNB1 | ADAMTS7 | SPARC |  | CORIN | TBCE |
| FN1 | ITGA1 | ECM1 | ADAMTS9 | SPP1 |  | CRTC2 | TBL1XR1 |
| GNG11 | ITGA2 | EMILIN1 | ADAMTSL1 | TGFB1 |  | CXXC5 | TLE3 |
| GSK3B | ITGA3 | ENTPD1 | ADAMTSL2 | TGFB2 |  | DCAF10 | TMEM230 |
| IGFBP4 | ITGAV | FBLN1 | ADAMTSL4 | TGFB3 |  | DDX3Y | TPP2 |
| IL1RN | ITGB1 | FN1 | ADAMTSL5 | THBS1 |  | DNAJC30 | WHRN |
| ILK | ITGB5 | ICAM1 | BMP1 | THBS2 |  | DUSP10 | ZFAND1 |
| ITGA5 | ITGB6 | ITGA2 | CD44 | THBS4 |  | ELOB | ZFP275 |
| ITGAV | ITGB8 | ITGA3 | COL10A1 | THSD1 |  | EMC3 | ZFP598 |
| ITGB1 | JUN | ITGA4 | COL1A1 | TIMP1 |  | FAM189B | ZFP991 |
| JAG1 | LOX | ITGA5 | COL1A2 | TIMP2 |  | FAM229B | ZSCAN12 |
| KRT14 | LTBP1 | ITGAL | COL3A1 | TIMP3 |  | FANCL |  |
| KRT7 | MMP13 | ITGAV | COL4A1 | TLL1 |  | GATAD2A |  |
| MAP1B | MMP14 | ITGB1 | COL4A2 | TNF |  | GGCT |  |
| MITF | MMP3 | ITGB2 | COL4A3 | TNFAIP1 |  | GGT1 |  |
| MMP3 | MMP8 | ITGB4 | COL4A3BP | TNFAIP2 |  | GM15417 |  |
| MMP9 | MMP9 | LAMA1 | COL4A4 | TNFRSF10B |  | GM6467 |  |
| MSN | MYC | LAMA2 | COL4A5 | TNFRSF11B |  | GM9392 |  |
| MST1R | NFKB1 | LAMA3 | COL5A1 | TNFRSF12A |  | GPR18 |  |
| NOTCH1 | PDGFA | LAMB2 | COL5A2 | TNFRSF13B |  | GRB14 |  |
| OCLN | PDGFB | LAMB3 | COL5A3 | TNFRSF14 |  | GTF2E1 |  |
| PDGFRB | PLAT | LAMC1 | COL7A1 | TNFRSF1A |  | H3F3A-PS1 |  |
| PLEK2 | PLAU | MMP11 | DNAJC4 | TNFRSF1B |  | HADHA |  |
| PTK2 | SERPINH1 | MMP12 | ELN | TNFRSF25 |  | HAVCR2 |  |
| PTP4A1 | SMAD2 | MMP13 | EMILIN1 | TNFRSF4 |  | HCFC2 |  |
| RAC1 | SMAD3 | MMP14 | EMILIN2 | TNFRSF9 |  | HIC2 |  |
| RGS2 | SMAD4 | MMP15 | F3 | TNFSF10 |  | HOXC9 |  |
| SERPINE1 | SMAD6 | MMP2 | FAS | TNFSF13 |  | HOXD4 |  |
| SMAD2 | SMAD7 | MMP3 | FBN1 | TNFSF15 |  | HSD17B12 |  |
| SNAI1 | SNAI1 | MMP7 | FBN2 | TNFSF9 |  | IARS |  |
| SPARC | SP1 | MMP8 | FN1 | VEGFA |  | IFI213 |  |
| STAT3 | STAT1 | MMP9 | HIF1A | VEGFB |  | IFT27 |  |
| STEAP1 | STAT6 | NCAM1 | HIF1AN | VEGFC |  | JAM2 |  |
| TCF3 | TGFB1 | POSTN | HYOU1 | YBX1 |  | JAM3 |  |
| TCF4 | TGFB2 | SELL | IL1B | ZCCHC2 |  | KATNBL1 |  |
| TFPI2 | TGFB3 | SELP | ITGA3 |  |  | KCNC3 |  |
| TGFB1 | TGFBR1 | SPARC | ITGAV |  |  | KCNH1 |  |
| TGFB2 | TGFBR2 | TGFBI | ITGB1 |  |  | KCNK6 |  |
| TGFB3 | TGIF1 | THBS2 | ITGB3 |  |  | KCTD10 |  |
| TMEFF1 | THBS2 | TIMP1 | LAMA1 |  |  | KLHDC2 |  |
| TMEM132A | TIMP2 | TIMP2 | LAMA2 |  |  | KPNA4 |  |
| TSPAN13 | TIMP3 | TIMP3 | LAMA3 |  |  | KPTN |  |
| TWIST1 | TNF | VCAM1 | LAMA4 |  |  | LGALS4 |  |
| VCAN | VEGFA |  | LAMB1 |  |  | LYZ2 |  |
| VIM |  |  | LAMB3 |  |  | MAD2L1BP |  |
| VPS13A |  |  | LAMC1 |  |  | MADD |  |
| WNT11 |  |  | LAMC2 |  |  | MFSD6L |  |
| WNT5A |  |  | LAMC3 |  |  | MTHFD2L |  |
| WNT5B |  |  | MEP1A |  |  | MYO1G |  |
| ZEB1 |  |  | MEP1B |  |  | NBR1 |  |
| ZEB2 |  |  | MGP |  |  | NCEH1 |  |

### Supplementary Table S1

Gene lists of gene lists with epithelial-to-mesenchymal transition (EMT), fibrosis and extracellular matrix (ECM) related genes, metzincins and related genes (MARGS), macrophage markers (MPh) and a list of random genes as a control. The fibrosis related gene lists was used for the principal component analysis (PCA) presented in figure 5B. All gene lists were used for the perturbation scores presented in figure 6. The gene lists for EMT, fibrosis and ECM originate from [SABiosciences](http://www.SABiosciences.com) as described in Methods. The lists of MARGS and macrophage markers originate from published articles (Marti et al., 2014, Mosser et al., 2008).
